# Fatiguing Joint Angle Does Not Influence Torque and Neuromuscular Responses Following Sustained, Isometric Forearm Flexion Tasks Anchored to Perceptual Intensity in Men

**DOI:** 10.3390/jfmk8030114

**Published:** 2023-08-10

**Authors:** Dolores G. Ortega, Terry J. Housh, Robert W. Smith, Jocelyn E. Arnett, Tyler J. Neltner, John Paul V. Anders, Richard J. Schmidt, Glen O. Johnson

**Affiliations:** 1 Exercise Physiology Laboratory, Department of Nutrition and Health Sciences, University of Nebraska—Lincoln, Lincoln, NE 68510, USA; dortega6@huskers.unl.edu (D.G.O.);; 2 The Exercise Science Program, Department of Human Sciences, The Ohio State University, Columbus, OH 43210, USA

**Keywords:** electromyography, mechanomyography, biceps brachii, isometric

## Abstract

This study examined the effects of joint angle (JA) on maximal voluntary isometric contraction (MVIC) and neuromuscular responses following fatiguing tasks anchored to RPE. Nine men (mean ± SD: age = 20.7 ± 1.2 yrs) performed forearm flexion MVICs at elbow JAs of 75° and 125° before and after sustained, isometric forearm flexion tasks to failure at fatiguing joint angles (FJA) of 75° and 125° anchored to RPE = 8. The amplitude and frequency of the electromyographic and mechanomyographic signals were recorded. Neuromuscular efficiency was calculated by dividing normalized torque by normalized electromyographic amplitude. A dependent *t*-test was used to assess the mean difference for time to task failure (TTF) between FJA. Repeated measure ANOVAs were used to assess mean differences for pre-test to post-test MVIC and neuromuscular responses. There was no significant difference between FJA for TTF (*p* = 0.223). The MVIC (collapsed across FJA and MVIC JA) decreased from pre-test to post-test (51.1 ± 5.0 vs. 45.3 ± 5.6 Nm, *p* < 0.001). Normalized neuromuscular parameters remained unchanged (*p* > 0.05). The FJA resulted in similar torque and neuromuscular responses, and the decreases in MVIC were not tracked by changes in the neuromuscular parameters. Thus, the neuromuscular parameters were not sensitive to fatigue, and pre-test to post-test measures may be compared between different FJA.

## 1. Introduction

Enoka and Stuart [1] have described fatigue as “…an acute impairment of performance that includes both an increase in the perceived effort necessary to exert a desired force and an eventual inability to produce this force” (p. 1631). To distinguish the two interdependent concepts of fatigue, Enoka and Duchateau [2] proposed the taxonomy of perceived fatigability and performance fatigability. Perceived fatigability is described as changes in the sensations experienced while performing a fatiguing task, while performance fatigability is defined as the reduction in an objective measure of performance, such as maximal voluntary isometric contraction (MVIC), across time [2]. Recent studies have assessed performance fatigability by using pre-test to post-test decreases in MVIC as a global measure of fatigue following sustained, isometric forearm flexion and leg extension tasks [3,4]. Exercise-induced processes occurring at or distal to the neuromuscular junction (i.e., peripheral fatigue) as well as proximal to the neuromuscular junction (i.e., central fatigue) can contribute to the global decrease in MVIC [5]. The mechanisms contributing to fatigue are dependent upon the nature of the task and the specific processes being stressed [6], therefore, using the appropriate measures to quantify fatigue allows for the identification of the mechanisms impairing performance [7].

Ratings of perceived exertion (RPE) are subjective measures that are influenced by the effort, strain, discomfort and/or fatigue experienced during exercise [8]. The OMNI-RES (0–10 point) RPE scale [9] has been applied to the RPE Clamp Model of Tucker [10] to prescribe exercise intensity, examine the interactions between perceived fatigability and performance fatigability, and assess fatigue-induced torque and neuromuscular responses following sustained, isometric forearm flexion and leg extension tasks anchored to a constant perceptual intensity [3,4,11,12,13,14]. Furthermore, to describe the neuromuscular responses to sustained, isometric tasks anchored to RPE, previous studies [3,4,11,12,13,14] have simultaneously examined the EMG and MMG signals. The amplitude (AMP) of the EMG signal reflects muscle activation [15], while the mean power frequency (MPF) is related to motor unit action potential conduction velocity [16]. Normalized EMG AMP has also been used to calculate neuromuscular efficiency (NME = normalized torque/normalized EMG AMP), which describes how the contractile elements of muscle respond to neural excitation [17]. Although there are differences of opinion regarding the applicability of NME [18], decreases in NME have been associated with excitation–contraction coupling failure resulting from peripheral fatigue [14,17,19]. With regard to the MMG signal, under some conditions, the AMP reflects motor unit recruitment, while the MPF qualitatively describes changes in the global firing rate of the activated, unfused motor units [20,21]. Therefore, changes in the EMG and MMG parameters from pre-test to post-test MVIC function as indirect, inferential measures that provide information about the motor unit activation strategies modulating fatigue-induced decreases in force production [3,4,12].

Recent studies have examined the effect of joint angle on the torque and neuromuscular responses during and following sustained, isometric forearm flexion tasks anchored to RPE [3,11,13,14]. It has become well accepted that MVIC force (i.e., torque) is influenced by the joint angle at which a fatiguing task is performed [22] due to differences in the overlap of actin and myosin affecting the ability to produce force [23]. In addition, Petrofsky and Phillips [24] have reported joint angle-specific differences in time to task failure (TTF) for sustained, isometric forearm flexion tasks with the greatest TTF at an elbow joint angle of 120°. Petrofsky and Phillips [24] have hypothesized that the differences in TTF were due to differences in the amount of actin and myosin overlapping at each elbow joint angle. Less is known, however, about the effects of joint angle on neuromuscular responses following fatiguing forearm flexion tasks anchored to RPE [3,11,14,24]. Weir et al. [25] have previously reported joint angle-specific differences in EMG and MMG responses between isometric dorsiflexion tasks anchored to force performed at 40° plantarflexion and 5° dorsiflexion in a combined sample of men and women. Arnett et al. [11] have recently reported joint angle-specific differences in performance fatigability and EMG AMP as well as anchoring scheme-specific differences in TTF in women who performed sustained, isometric forearm flexion tasks anchored to RPE = 8 and the torque value that corresponded to RPE = 8 at fatiguing joint angles of 75° and 125°. Hunter [26] has stated that there are sex differences in performance fatigability following fatiguing isometric tasks, with less performance fatigability observed in women compared with men. Differences in muscle mass and strength, blood flow and muscle perfusion, muscle fiber contractile function and properties, skeletal muscle metabolism, and voluntary activation contribute to the sex differences in performance fatigability [27]. Furthermore, the magnitude of the sex differences is dependent on the nature of the task [26]. Thus, although joint angle has been shown to affect MVIC torque and neuromuscular parameters in a combined sample of men and women [25], as well as in women only [11], it remains unclear whether these responses are similar when a fatiguing task is anchored to a constant perceptual intensity in men. Therefore, the purpose of this study was to examine the effects of joint angle on the torque and neuromuscular responses following sustained, isometric forearm flexion tasks anchored to RPE = 8 (OMNI-RES scale) at fatiguing joint angles of 75° (FJA75) and 125° (FJA125) in men. Based on prior studies that utilized the RPE Clamp Model [10] during fatiguing forearm flexion tasks [3,11], it was hypothesized that: (1) torque would decrease from pre-test to post-test MVIC; (2) MVIC torque would be affected by the joint angle where the MVICs were assessed but not the fatiguing joint angle of the sustained task; (3) there would be joint angle-specific fatigue-induced decreases in EMG AMP; (4) there would be fatigue-induced decreases in EMG MPF from pre-test to post-test MVIC with no differences between joint angles; and (5) there would be no fatigue-induced changes in MMG AMP or MMG MPF from pre-test to post-test MVIC.

## 2. Materials and Methods

### 2.1. Subjects

An a priori sample size calculation (G*Power version 3.1.9.4, Düsseldorf, Germany) using previously reported performance fatigability data [3] identified that 8 subjects were required to demonstrate mean differences between 2 dependent groups using repeated measures ANOVAs, an effect size of $\eta_{p}^{2}$ = 0.752, a power of 0.95, and an alpha of 0.05. Nine men (mean ± SD: age = 20.7 ± 1.2 yrs, height = 182.4 ± 5.7 cm, body mass = 84.9 ± 15.3 kg) volunteered for the present study. The subjects were recreationally active and participated in resistance and/or aerobic exercise for a minimum of 3 d∙wk^−1^ [28]. All testing was performed using the right arm, so subjects were required to be right hand dominant based on throwing preference [29] and free of upper body pathologies that would affect performance. Six out of the nine subjects indicated that they had been using supplements, including protein and creatine, within the last six weeks. The subjects were asked to maintain their normal supplementation use throughout the duration of the study. The subjects in the current study were part of a large multi-independent and dependent variable study, however, none of these data have been previously published [3,11]. The study was approved by the University Institutional Review Board for Human Subjects (IRB Approval #: 20201220785FB).

### 2.2. Time Course of Proceduresv

The subjects signed a written informed consent document and completed a Health History Questionnaire prior to testing. Each subject visited the laboratory on three separate occasions, including an orientation session and two testing visits, separated by 24 to 144 h. Demographic information was recorded on the initial visit as part of the orientation session and the subjects were familiarized with the standardized warm-up, testing protocol, and the standardized OMNI-RES [9] instructions. During the testing visits, the subjects completed the standardized warm-up, pre-test and post-test MVICs at elbow joint angles of 75° (MVIC JA75) and 125° (MVIC JA125), and a sustained, isometric forearm flexion task to failure anchored to RPE = 8 at FJA75 or FJA125. The EMG and MMG signals were recorded from the biceps brachii (BB) muscle of the right arm throughout the sustained tasks and all MVIC testing. The time course of procedures is presented in Table 1.

### 2.3. OMNI-RES Scale Standardized Anchoring Instructions

The anchoring instructions in this study were originally developed by Gearhart Jr. et al. [30] as a standardized method to gauge training intensity during lower body exercise. The instructions were modified by Smith et al. [13] for use during isometric forearm flexion tasks. To promote the proper use of the OMNI-RES scale, the following standardized anchoring instructions were read to each subject during the orientation session and prior to each sustained task anchored to RPE = 8: “You will be asked to set an anchor point for both the lowest and highest values on the perceived exertion scale. To set the lowest anchor, you will be asked to lay quietly without contracting your forearm flexor muscles to familiarize yourself with an RPE of zero. Following this, you will be asked to perform a maximal voluntary isometric contraction to familiarize yourself with an RPE of 10. When instructed to match a perceptual value corresponding to the OMNI-RES scale, perceived exertion should be relative to these defined anchors”.

### 2.4. Orientation Session

**Figure 1:**
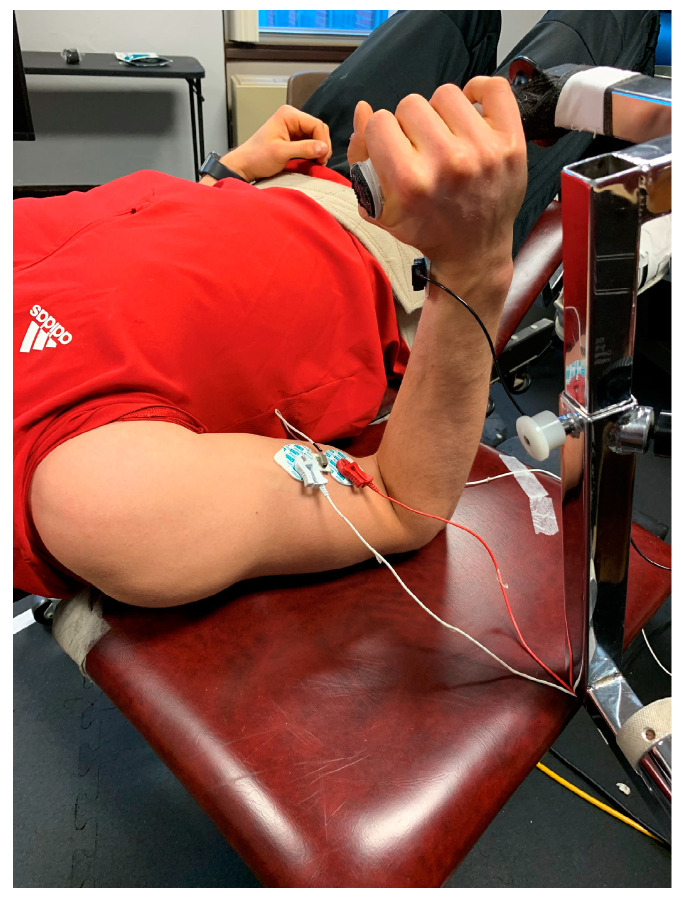
Experimental setup on Cybex II isokinetic dynamometer.

During the orientation session (Table 1), the subject’s age, height, and body mass were recorded. The subject was then oriented to their testing position on an upper body exercise table (UBXT) with the lateral epicondyle of the humerus of the dominant arm aligned with the lever arm of the isokinetic dynamometer, in accordance with the Cybex II (Cybex II International Inc. Medway, MA) user’s manual (Figure 1). Once positioned, subjects were familiarized with the OMNI-RES (0–10) RPE scale [9] and read the standardized OMNI-RES (0–10) RPE instructions [9,13]. Robertson et al. [9] have shown the OMNI-RES (0–10) RPE scale to be valid and reliable for the quantification of perception during resistance exercise. The subjects then completed the standardized warm-up, 2, 3 s isometric forearm flexion MVICs to set a perceptual anchor corresponding to RPE = 10, and a brief (approximately 1 min) sustained, isometric forearm flexion task anchored to RPE = 8 to become familiarized with the testing and anchoring procedures.

### 2.5. Testing Visit

During the testing visits (Table 1), the subjects were again positioned on the UBXT and performed the standardized warm-up followed by 1 min of rest. The subject was then read the OMNI-RES instructions relating to the anchoring procedures and performed 2, 3 s forearm flexion pre-test MVICs on a calibrated Cybex II dynamometer at MVIC JA75 and MVIC JA125, in a randomized order. Strong verbal encouragement was provided during each MVIC trial. After the pre-testing MVICs, the subject performed the sustained, isometric forearm flexion task anchored to RPE = 8 (OMNI-RES scale) at either FJA75 or FJA125. Due to differences in the overlap of actin–myosin binding throughout the range of motion [23], FJA75 and FJA125 were selected to reflect a range of isometric torque production [22]. During the sustained tasks anchored to RPE, the subjects were unaware of torque and elapsed time to avoid pacing strategies [12]. In addition, the subjects were asked what their RPE was every 30 s to assure compliance with RPE = 8. The trials were sustained to task failure, which was defined as a torque that would require RPE > 8, or the torque was reduced to zero. The subjects were continuously advised to be attentive to sensations such as strain, intensity, discomfort, and fatigue felt during the sustained task to maintain appropriate levels of exertion [9] and to relate the levels of exertion to the previously set anchors. Thus, the subjects were free to decrease torque to maintain a constant RPE value. At task failure, the TTF was recorded and post-test MVICs were performed in a manner identical to the pre-test MVICs.

### 2.6. Electromyographic, Mechanomyographic, and Torque Acquisition

During all testing visits, pre-gelled surface EMG electrodes (Ag/AgCl, Accusensor; Lynn Medical, Wixom, MI, USA) were placed in a bipolar arrangement (30 mm center-to-center) on the BB of the dominant arm in accordance with the recommendations of the Surface Electromyography for the Non-Invasive Assessment of Muscles [31]. Prior to electrode placement, the skin was carefully shaved, abraded, and cleaned with alcohol. The electrodes were placed between the medial acromion and the antecubital fossa, at one-third the distance from the antecubital fossa over the BB. A reference electrode was placed on the radial styloid process of the forearm. A miniature accelerometer (Entras EGAS FT 10, bandwidth 0–200 Hz, dimensions 1.0 × 1.0 × 0.5 cm, mass 1.0 g, sensitivity 550.4 mV∙g^−1^) was placed between the bipolar EMG electrodes using double-sided adhesive tape (Figure 1).

The raw EMG and MMG signals were digitized at 2000 samples per second using a 12-bit analog-to-digital converter (Model MP150; Biopac Systems, Inc., Goleta, CA, USA). The EMG and MMG signals were then stored on a personal computer (Acer Aspire TC-895-UA91 Acer Inc., San Jose, CA, USA) for signal processing that was performed using custom programs written with LabVIEW programming software (version 20.0f1, National Instruments, Austin, TX, USA). The EMG and MMG signals were digitally band-pass filtered (fourth-order Butterworth) at 10–500 Hz and 5–100 Hz, respectively. A 1 s epoch from the center of the highest 3 s forearm flexion MVIC was used to calculate the AMP (root mean square) for EMG (µVrms) and MMG (m∙s^−2^) signals and the mean power frequency (MPF in Hz) for both signals. The MPF was chosen to reflect the power density spectrum and was calculated as described by Kwatny et al. [32]. The NME was calculated by dividing normalized torque by normalized EMG AMP [17,33]. The torque signals were sampled from the digital torque of the Cybex II isokinetic dynamometer and stored on a personal computer (Acer Aspire TC-895-UA91 Acer Inc., San Jose, CA, USA) for analysis.

### 2.7. Statistical Analysis

The test–retest reliability for the MVICs, EMG AMP, EMG MPF, MMG AMP, and MMG MPF at MVIC JA75 and MVIC JA125 were assessed with a repeated measures ANOVA to evaluate systematic error and a 2,1 model was used to determine the intraclass correlation coefficient (ICC) [34]. The mean differences for pre-test versus post-test MVICs, normalized (% pre-test MVIC) neuromuscular parameters (EMG AMP, EMG MPF, MMG AMP, and MMG MPF), and NME were analyzed with six, separate 2 (fatiguing joint angle: 75° vs. 125°) × 2 (time: pre-test vs. post-test) × 2 (MVIC joint angle: 75° vs. 125°) repeated measures ANOVAs. Significant interactions were decomposed with follow-up post-hoc, Bonferroni corrected, paired *t*-tests [34]. The mean difference for the TTF was determined using a dependent *t*-test. Effect sizes were reported as partial eta squared ($\eta_{p}^{2}$) for the ANOVAs and Cohen’s *d* for the pairwise comparisons. All statistical analyses were completed in IBM SPSS v. 28 (Armonk, NY, USA).

## 3. Results

The pre-test and post-test values for MVIC, EMG AMP, EMG MPF, MMG AMP, MMG MPF, and NME are presented by joint angle in Table 2.

### 3.1. Reliability

Table 3 includes the test–retest reliability parameters (*p*-value (systematic error), ICC, ICC_95%_, and SEM) for MVIC, EMG AMP, EMG MPF, MMG AMP, and MMG MPF. There were no mean differences (*p* > 0.05) for test versus retest for MVIC and the neuromuscular parameters. The ICC values ranged from 0.025 (EMG MPF at MVIC JA125) to 0.829 (EMG MPF MVIC JA75).

### 3.2. Time to Task Failure

The results of the dependent *t*-test for TTF indicated no significant mean difference between FJA75 and FJA125 (276.3 ± 185.0 vs. 227.7 ± 96.4 s, *p* = 0.223, *d* = 0.440).

### 3.3. Maximal Voluntary Isometric Contraction

**Figure 2:**
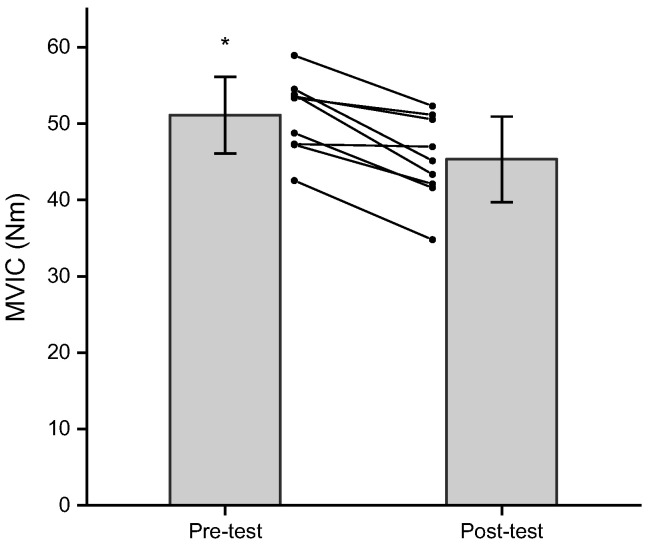
Pre- and post-test (mean ± SD) MVIC values (collapsed across fatiguing joint angle and MVIC joint angle). Spaghetti graphs are the individual subject responses. * Pre-test > post-test (*p* < 0.001).

The results of the three-way repeated measures ANOVA for MVIC indicated no significant three-way interaction (*p* = 0.789, $\eta_{p}^{2}$ = 0.009), two-way interactions (*p* = 0.735–0.992, $\eta_{p}^{2}$ = 0.000–0.015) or main effect for fatiguing joint angle (*p* = 0.173, $\eta_{p}^{2}$ = 0.218), but there were significant main effects for time (*p* < 0.001, $\eta_{p}^{2}$ = 0.766) and MVIC joint angle (*p* = 0.041, $\eta_{p}^{2}$ = 0.427). The main effect for time (collapsed across fatiguing joint angle and MVIC joint angle) indicated that the pre-test MVIC was significantly greater than the post-test MVIC (51.1 ± 5.0 vs. 45.3 ± 5.6 Nm, *p* < 0.001, *d* = 1.708; Bonferroni corrected alpha = 0.025) (Figure 2). The follow-up pairwise comparison for the main effect for MVIC joint angle (collapsed across fatiguing joint angle and time) indicated no difference between MVIC JA75 and MVIC JA125 (46.0 ± 6.3 vs. 50.5 ± 5.2 Nm, *p* = 0.041, *d* = −0.814; Bonferroni corrected alpha = 0.025).

### 3.4. Electromyographic Amplitude

**Figure 3:**
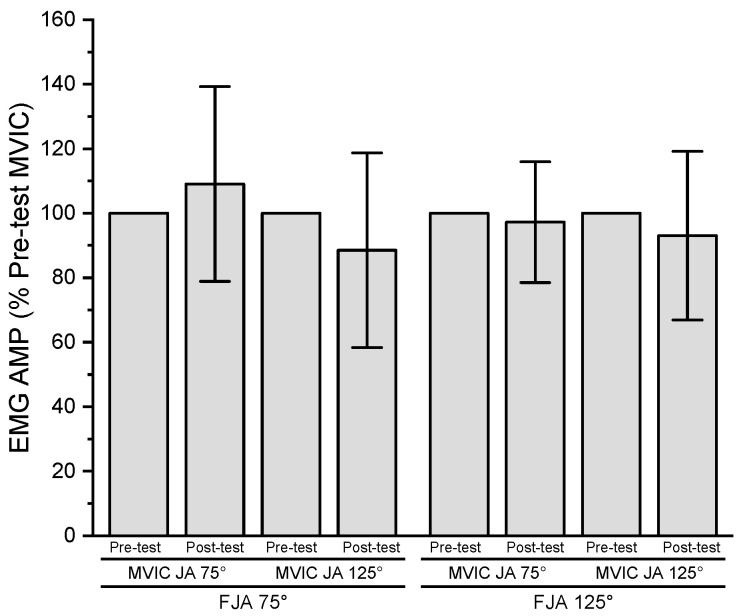
Pre- and post-test (mean ± SD) normalized EMG AMP values for MVIC joint angle 75° (MVIC JA 75°) and 125° (MVIC JA 125°) at fatiguing joint angle 75° (FJA 75°) and 125° (FJA 125°).

The results of the three-way repeated measures ANOVA for normalized EMG AMP indicated no significant three-way interaction (*p* = 0.428, $\eta_{p}^{2}$ = 0.080), two-way interactions (*p* = 0.220–0.654, $\eta_{p}^{2}$ = 0.026–0.181), or main effects for fatiguing joint angle (*p* = 0.654, $\eta_{p}^{2}$ = 0.026), time (*p* = 0.498, $\eta_{p}^{2}$ = 0.059), or MVIC joint angle (*p* = 0.220, $\eta_{p}^{2}$ = 0.181) (Figure 3).

### 3.5. Electromyographic Mean Power Frequency

**Figure 4:**
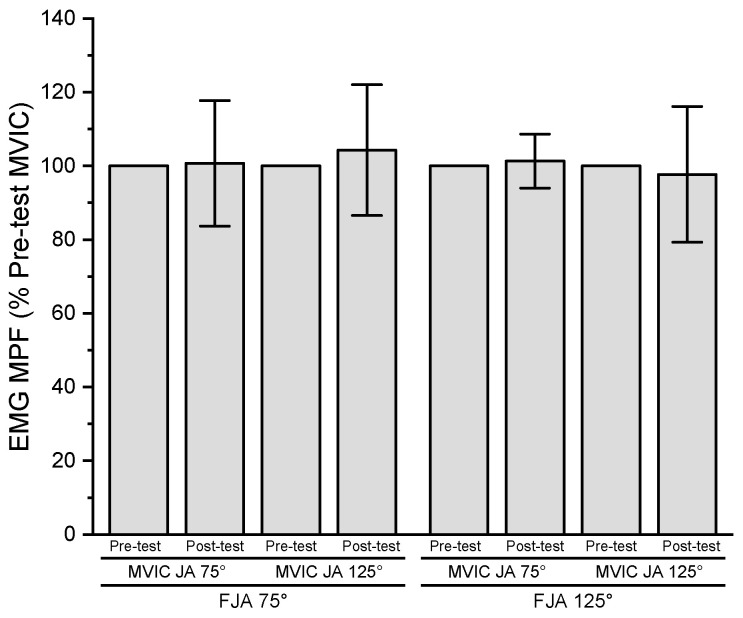
Pre- and post-test (mean ± SD) normalized EMG MPF values for MVIC joint angle 75° (MVIC JA 75°) and 125° (MVIC JA 125°) at fatiguing joint angle 75° (FJA 75°) and 125° (FJA 125°).

The results of the three-way repeated measures ANOVA for normalized EMG MPF indicated no significant three-way interaction (*p* = 0.556, $\eta_{p}^{2}$ = 0.045), two-way interactions (*p* = 0.556–0.998, $\eta_{p}^{2}$ = 0.000 to $\eta_{p}^{2}$ = 0.045), or main effects for fatiguing joint angle (*p* = 0.612, $\eta_{p}^{2}$ = 0.034), time (*p* = 0.754, $\eta_{p}^{2}$ = 0.013), or MVIC joint angle (*p* = 0.998, $\eta_{p}^{2}$ = 0.000) (Figure 4).

### 3.6. Mechanomyographic Amplitude

**Figure 5:**
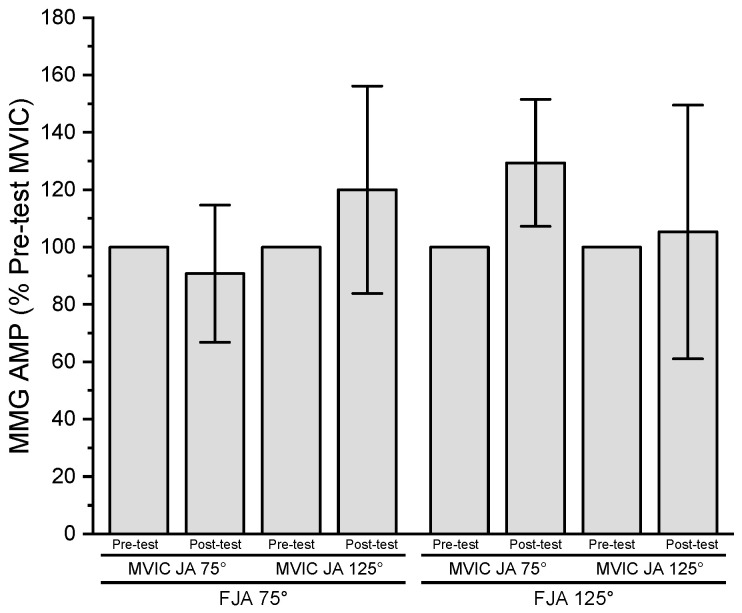
Pre- and post-test (mean ± SD) normalized MMG AMP values for MVIC joint angle 75° (MVIC JA 75°) and 125° (MVIC JA 125°) at fatiguing joint angle 75° (FJA 75°) and 125° (FJA 125°).

The results of the three-way repeated measures ANOVA for normalized MMG AMP indicated no significant three-way interaction (*p* = 0.061, $\eta_{p}^{2}$ = 0.373), two-way interactions (*p* = 0.061–0.824, $\eta_{p}^{2}$ = 0.007 to $\eta_{p}^{2}$ = 0.373), or main effects for fatiguing joint angle (*p* = 0.168, $\eta_{p}^{2}$ = 0.223), time (*p* = 0.092, $\eta_{p}^{2}$ = 0.314), or MVIC joint angle (*p* = 0.824, $\eta_{p}^{2}$ = 0.007) (Figure 5).

### 3.7. Mechanomyographic Mean Power Frequency

**Figure 6:**
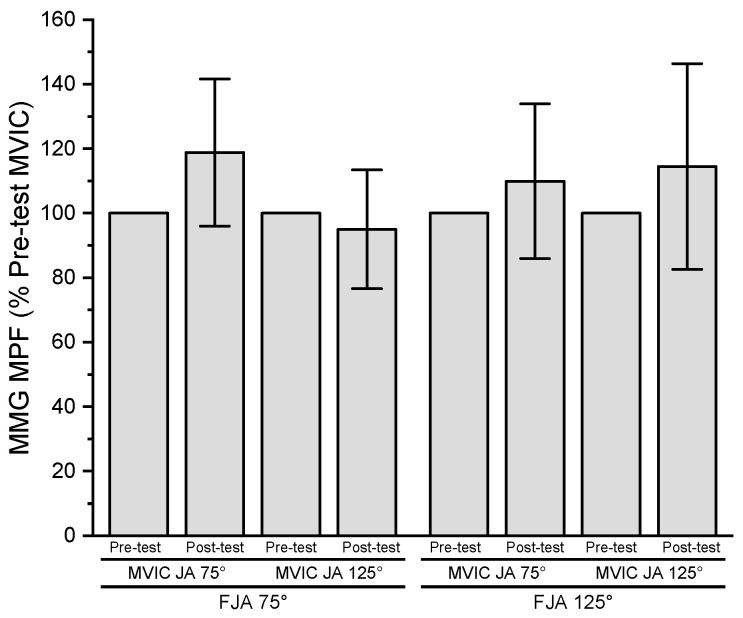
Pre- and post-test (mean ± SD) normalized MMG MPF values for MVIC joint angle 75° (MVIC JA 75°) and 125° (MVIC JA 125°) at fatiguing joint angle 75° (FJA 75°) and 125° (FJA 125°).

The results of the three-way repeated measures ANOVA for normalized MMG MPF indicated no significant three-way interaction (*p* = 0.161, $\eta_{p}^{2}$ = 0.230), two-way interactions (*p* = 0.161–0.453, $\eta_{p}^{2}$ = 0.072–0.230), or main effects for fatiguing joint angle (*p* = 0.453, $\eta_{p}^{2}$ = 0.072), time (*p* = 0.066, $\eta_{p}^{2}$ = 0.361), or MVIC joint angle (*p* = 0.258, $\eta_{p}^{2}$ = 0.156) (Figure 6).

### 3.8. Neuromuscular Efficiency

**Figure 7:**
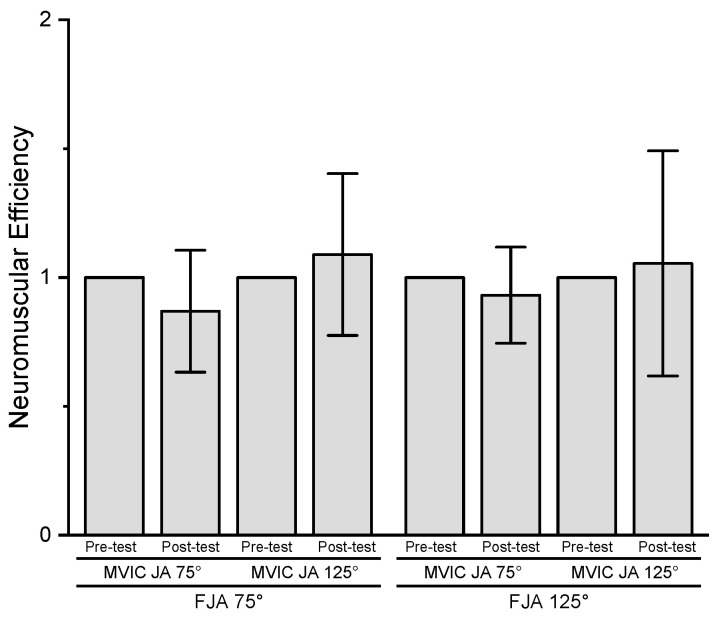
Pre- and post-test (mean ± SD) neuromuscular efficiency (NME: normalized torque/normalized EMG AMP) values for MVIC joint angle 75° (MVIC JA 75°) and 125° (MVIC JA 125°) at fatiguing joint angle 75° (FJA 75°) and 125° (FJA 125°).

The results of the three-way repeated measures ANOVA for NME indicated no significant three-way interaction (*p* = 0.693, $\eta_{p}^{2}$ = 0.021), two-way interactions (*p* = 0.096–0.863, $\eta_{p}^{2}$ = 0.004–0.308), or main effects for fatiguing joint angle (*p* = 0.863, $\eta_{p}^{2}$ = 0.004), time (*p* = 0.813, $\eta_{p}^{2}$ = 0.007), or MVIC joint angle (*p* = 0.096, $\eta_{p}^{2}$ = 0.308) (Figure 7).

## 4. Discussion

The test–retest reliability analyses for the MVIC and neuromuscular parameters (EMG AMP, EMG MPF, MMG AMP, and MMG MPF) in the current study are presented in Table 3. There were no significant mean differences for the test versus retest for forearm flexion MVIC and the ICCs ranged from R = 0.388–0.559. These ICCs reflected poor to fair reliability [35] and were lower than those previously reported for MVIC by Hill et al. [36] and Arnett et al. [11]. There were also no significant mean differences between the test and retest reliability for EMG AMP, EMG MPF, MMG MPF, and MMG AMP. The ICCs for the neuromuscular parameters ranged from R = 0.025–0.829 and reflected poor to excellent reliability [35]. The ICCs reported for the neuromuscular parameters in this study were comparable to those previously reported for EMG AMP and EMG MPF by Arnett et al. [11] (R = 0.406–0.743), but lower than those previously reported for EMG AMP, EMG MPF, MMG AMP, and MMG MPF by Hill et al. [36] (R = 0.863–0.975). Koo and Li [37] have stated that ICCs can be affected by the degree of variability of the sample. In addition, a slight day-to-day change in the location of the electrodes and accelerometer used to record the EMG and MMG signals during the MVICs may have affected the absolute values of the neuromuscular parameters [38] that were used in the reliability analyses. In this study, the fatiguing tasks at FJA75 and FJA125 were performed on separate days and, therefore, required the removal and replacement of the electrodes and accelerometer onto the skin’s surface. To analyze the mean differences of the neuromuscular parameters across testing visits, however, the values for each subject were normalized to the individual’s highest MVIC at each specific visit and the responses were expressed as a percentage of the MVIC [38]. Halaki and Ginn [39] have stated that normalization procedures utilizing MVICs can be used to reliably compare the neuromuscular responses between tasks and between subjects.

The present findings indicate no joint angle-specific difference in MVIC values (MVIC JA75 = 46.0 ± 6.3 Nm vs. MVIC JA125 = 50.5 ± 5.2 Nm). Typically, during forearm flexion muscle actions, the highest MVIC values occur at elbow joint angles of approximately 90–120° [22,24,40,41,42,43] due to optimal actin–myosin overlap and formation of cross-bridge attachments [23,24,44]. At elbow joint angles closer to full flexion and extension, however, the disadvantageous overlap of actin and myosin interferes with the formation of cross-bridge attachments and impairs force production [23,24,44]. Near full flexion, there is excessive overlap of actin and myosin, and near full extension, there is insufficient overlap of actin and myosin [23,24,44]. The elbow joint angles assessed in the current study are not within the optimal range, which may explain the lack of difference in MVIC.

In the current study, there were similar declines in MVIC torque for the two MVIC joint angles (75° = 11.5% and 125° = 11.0%) from pre-test to post-test for both fatiguing joint angles (FJA75 and FJA125) (Table 2). Previous studies have applied the RPE Clamp Model [10] to sustained, isometric forearm flexion tasks anchored to RPE = 8 at a fatiguing joint angle of 100° in men [3] and fatiguing joint angles of 75° and 125° in women [11]. The decreases in MVIC torque in the present study were similar to those in men at a fatiguing joint angle of 100°, which ranged from 9.9 to 20.7% [3]. In contrast with the current study, Arnett et al. [11] have reported greater decreases in MVIC torque in women at fatiguing joint angles of 75° and 125° that ranged from 15.3 to 23.7%. Pre-test to post-test declines in MVIC torque have been used as a global measure of performance fatigability [2]. Thomas et al. [45] have hypothesized that the magnitude of performance fatigability is directly related to the amount of engaged muscle mass during the task. Specifically, when fatiguing tasks are performed at the same relative intensity, a smaller amount of engaged muscle mass results in greater performance fatigability due to increased tolerance to localized metabolic perturbations as well as reduced disruption to the cardiac and respiratory systems [45]. Thus, the dissimilarities in performance fatigability between the men in the present study and the women in Arnett et al. [11] may have been due to differences in the amount of engaged muscle mass during the fatiguing tasks. Future studies should assess the effects of sex and muscle mass on the decline in MVIC torque following sustained tasks anchored to RPE.

The results of the current study indicated that there was no significant difference (*p* = 0.223, *d* = 0.440) between the fatiguing joint angles for the TTF (FJA75 = 276.3 ± 185.0 s vs. FJA125 = 227.7 ± 96.4 s). These findings are consistent with those of Smith et al. [14] who also reported no difference in TTF (FJA75 = 521.8 ± 327.2 s vs. FJA125 = 572.7 ± 333.2 s) during sustained, isometric forearm flexion tasks anchored to RPE = 7 in men. Petrofsky and Phillips [24] have reported an inverse relationship between isometric force production and TTF. Therefore, the lack of difference in TTF in the current study and that of Smith et al. [14] was likely due to the tasks being held at one RPE value for each of the fatiguing joint angles. Future studies should continue to examine how anchoring sustained, isometric tasks to different RPE values influences TTF.

The present findings indicate that, regardless of the fatiguing joint angle or MVIC joint angle, there were no changes in normalized EMG AMP, EMG MPF, MMG AMP, MMG MPF, or NME from pre-test to post-test. The fatigue-induced changes in EMG AMP as an indicator of muscle activation [15] and EMG MPF as an indicator of motor unit action potential conduction velocity [16] have been used to derive inferences regarding the contributions of peripheral and central mechanisms to performance fatigability [46]. Peripheral mechanisms, which occur at or distal to the neuromuscular junction [5] are affected by the fatigue-induced accumulation of metabolic by-products [47] and acidification of the cellular environment [48]. Specifically, the accumulation of inorganic phosphate (P_i_), intramuscular hydrogen ions (H^+^), and extracellular potassium (K^+^) as well as the decrease in pH lead to disruptions in action potential propagation, excitation–contraction coupling, actin–myosin binding, Ca^2+^ release and reuptake by the sarcoplasmic reticulum [48,49], and troponin–calcium binding [49]. Fatigue-induced build-up of H^+^ in the interstitial space of skeletal muscle can be sensed by group III/IV afferents and contribute to central mechanisms of fatigue, including reduced central motor command and decreased motor unit recruitment [50]. Previous studies have reported increases [11], decreases [3,4,11,12,51,52], or no changes [3,4,12,51,52] in EMG AMP and/or EMG MPF during MVICs measured prior to and following fatiguing isometric tasks. It had been suggested that the increases in EMG AMP were due to synchronization [11], the decreases in EMG AMP and EMG MPF were due to the accumulation of metabolic byproducts [3,4,12,51], and the lack of changes in EMG AMP were due to the maintenance of muscle activation [4,12,51,52]. Neuromuscular efficiency [14,33], used to describe the response of the contractile elements of muscle to neural excitation [17], may be representative of peripheral and/or central mechanisms of fatigue. If both normalized MVIC torque and normalized EMG AMP decrease, the NME ratio could remain constant and be an indicator of central fatigue [50]. In contrast, decreases in MVIC torque with no changes in EMG AMP could result in decreased NME and be an indicator of peripheral fatigue due to excitation–contraction coupling failure [53]. For both fatiguing joint angles in the current study, there were no changes in EMG AMP, EMG MPF, or NME from pre-test to post-test at each MVIC joint angle (75° and 125°). The present results were not in agreement with previous studies that anchored sustained, isometric forearm flexion tasks to RPE [3,11]. In men, a sustained task anchored to RPE = 8 at a fatiguing joint angle of 100° resulted in MVIC joint angle-specific differences in EMG AMP, but no changes in EMG MPF [3]. In women, sustained tasks anchored to RPE = 8 performed at fatiguing joint angles of 75° and 125° resulted in joint angle-specific differences in EMG AMP and parallel decreases in EMG MPF [11]. Thus, the unique nature of fatiguing tasks anchored to RPE, with the ability to voluntarily reduce torque, results in different neuromuscular responses than tasks anchored to force. It is possible that, in the present study, the EMG responses were not sensitive to the magnitudes of performance fatigability that were demonstrated.

The MMG signal has been described as the mechanical counterpart of motor unit electrical activity reflected in the EMG signal [20,25], and has been used to monitor fatigue from pre-test to post-test MVICs, as well as to derive inferences regarding fatigue-induced changes in motor unit activation strategies [3,4,51,52]. The MMG signal reflects the lateral oscillations of activated muscle fibers and, under some conditions, MMG AMP and MMG MPF track changes in motor unit recruitment and the global firing rate of unfused, activated motor units, respectively [20,21]. Both domains, however, can be affected by muscle stiffness and intramuscular fluid pressure [20,21] and be representative of the mechanical factors affecting the MMG signal rather than the motor unit activation strategies. Previous studies examining MMG responses following fatiguing intermittent and sustained isometric tasks have reported increases [51], decreases [52], or no changes [3,4,51,52] in MMG AMP and/or MMG MPF. It was hypothesized that the increased MMG AMP was due to decreased muscle stiffness [51], the decreased MMG MPF was due to the decreased global firing rate of unfused, activated muscle fibers [52], and the lack of changes in MMG AMP and MMG MPF were due to the fatiguing tasks having no effect on motor unit recruitment and/or firing rate [3,4]. The present findings indicated no changes in MMG AMP or MMG MPF from pre-test to post-test at each MVIC joint angle (75° and 125°) for both fatiguing joint angles (FJA75 and FJA125). The results of the current study were consistent with those of Arnett et al. [3], where a sustained, isometric forearm flexion task anchored to RPE = 8 at a fatiguing joint angle of 100° in men resulted in no pre-test to post-test differences in MMG AMP or MMG MPF. Therefore, sustained tasks anchored to a high perceptual intensity are characterized by distinctive neuromuscular responses associated with the freedom to reduce torque that do not track the decreases in performance fatigability.

A limitation of the current study included the use of neuromuscular parameters as indirect indicators of fatigue-induced changes in motor unit activation strategies and mechanisms contributing to performance fatigability. Future studies should use the interpolated twitch technique and the potentiated twitch amplitude procedures to examine the contributions of central and peripheral mechanisms of fatigue during tasks anchored to RPE. Furthermore, future studies may consider assessing fatigue following sustained tasks anchored to RPE using biochemical markers, such as creatine kinase, cortisol, and blood lactate. In addition, the present findings were limited to the acute neuromuscular responses from the BB following sustained, isometric forearm flexion tasks anchored to a high, constant RPE value at fatiguing joints angles of 75° and 125° in college-aged men. Future studies should sample signals simultaneously from the three muscles that contribute to forearm flexion (i.e., BB, brachioradialis, and brachialis), anchor the fatiguing tasks to different RPE values, examine the effects of different joint angles, consider the chronic effects of fatigue, and replicate the results in women and men of a wider age range.

## 5. Conclusions

In summary, the present findings indicated that the sustained, isometric forearm flexion tasks anchored to RPE = 8 resulted in similar MVIC torque decreases between FJA75 and FJA125 as well as the two MVIC joint angles (75° and 125°). The neuromuscular parameters (EMG AMP, EMG MPF, MMG AMP, MMG MPF, and NME) used in the present study to derive inferences regarding fatigue-induced changes in motor unit activation strategies and mechanisms of fatigue, however, did not change from pre-test to post-test MVICs and were dissociated from the decreases in MVIC torque. Thus, there were no fatiguing joint angle-specific differences in torque or neuromuscular responses following sustained, isometric tasks anchored to RPE. Furthermore, due to the unique nature of sustained tasks anchored to RPE, torque was free to decrease to maintain the high perceptual intensity. Therefore, it is possible that, under some conditions, the EMG and MMG signals may not be sensitive to fatigue that results in reduced torque from pre-test to post-test MVICs.

## Figures and Tables

**Table 1:** Time course of procedures.

Orientation Session	Testing Visits 1 and 2
Informed Consent. Health History Questionnaire. Age, height, and body mass recorded. Familiarization with testing procedures. Read the standardized anchoring instructions (OMNI-RES scale). Standardized warm-up: 4, 3 s submaximal (50–75% of max effort) isometric forearm flexion muscle actions. Isometric forearm flexion MVICs (2 repetitions, 3 s) to set a perceptual anchor of RPE = 10. Brief (~1 min) sustained, isometric forearm flexion task anchored to RPE = 8.	Standardized warm-up. Read the standardized anchoring instructions (OMNI-RES scale). Pre-test: 2, 3 s MVICs at joint angles of 75° and 125°, in a randomized order. Sustained, isometric forearm flexion task anchored to RPE = 8 (OMNI-RES scale) performed at fatiguing joint angles of 75° and 125° to task failure, in a randomized order. Post-test: 2, 3 s MVICs at joint angles of 75° and 125°, in a randomized order.

MVIC = maximal voluntary isometric contraction; RPE = rating of perceived exertion.

**Table 2:** Absolute mean ± SD values for maximal voluntary isometric contraction (MVIC) torque, electromyographic amplitude (EMG AMP), electromyographic mean power frequency (EMG MPF), mechanomyographic amplitude (MMG AMP), mechanomyographic mean power frequency (MMG MPF), and neuromuscular efficiency (NME) prior to (pre-test) and following (post-test) the sustained isometric forearm flexion tasks to failure anchored to RPE = 8.

	Fatiguing Joint Angle 75°		Fatiguing Joint Angle 125°	
	Pre-Test	Post-Test	Pre-Test	Post-Test
MVIC (Nm)				
Joint Angle				
75°	50.0 ± 8.0	44.4 ± 7.6	47.5 ± 6.1	41.9 ± 8.0
125°	54.9 ± 6.7	48.6 ± 7.2	52.0 ± 5.9	46.5 ± 5.8
EMG AMP (µVrms)				
Joint Angle				
75°	987.0 ± 334.8	1035.9 ± 365.0	1195.6 ± 501.4	1123.9 ± 439.9
125°	1198.5 ± 551.0	954.4 ± 317.7	976.7 ± 495.0	885.1 ± 507.6
EMG MPF (Hz)				
Joint Angle				
75°	71.4 ± 13.7	71.4 ± 15.7	73.6 ± 11.8	74.4 ± 12.1
125°	67.0 ± 8.5	69.4 ± 11.5	74.0 ± 15.4	70.5 ± 11.0
MMG AMP (m∙s^−2^)				
Joint Angle				
75°	0.48 ± 0.19	0.43 ± 0.16	0.37 ± 0.19	0.47 ± 0.23
125°	0.49 ± 0.17	0.56 ± 0.14	0.47 ± 0.17	0.45 ± 0.17
MMG MPF (Hz)				
Joint Angle				
75°	21.9 ± 6.0	26.1 ± 8.1	22.5 ± 5.6	25.0 ± 9.2
125°	25.9 ± 6.1	24.8 ± 8.4	26.7 ± 8.8	28.3 ± 6.0
NME				
Joint Angle				
75°	1.00 ± 0.00	0.87 ± 0.24	1.00 ± 0.00	0.93 ± 0.19
125°	1.00 ± 0.00	1.08 ± 0.31	1.00 ± 0.00	1.05 ± 0.44

**Table 3:** Reliability data for maximal voluntary isometric contraction (MVIC) torque and neuromuscular parameters (EMG AMP, EMG MPF, MMG AMP, and MMG MPF) during the pre-test forearm flexion MVICs at elbow joint angles (JA) of 75° and 125°.

**MVIC (mean ± SD)**	**Visit 1**	**Visit 2**	**P**	**ICC**	**ICC_95%_**
Forearm flexion at JA75 (Nm)	49.1 ± 8.6	48.4 ± 5.6	0.771	0.559	−0.164–0.882
Forearm flexion at JA125 (Nm)	54.2 ± 6.3	52.7 ± 6.6	0.574	0.388	−0.365–0.822
**Neuromuscular Parameters (mean ± SD)**					
EMG AMP at JA75 (µVrms)	989.7 ± 342.5	1194.0 ± 497.5	0.222	0.393	−0.237–0.814
EMG AMP at JA125 (µVrms)	1174.7 ± 557.8	1000.5 ± 498.0	0.332	0.540	−0.100–0.872
EMG MPF at JA75 (Hz)	71.7 ± 14.5	73.2 ± 10.9	0.551	0.829	0.425–0.959
EMG MPF at JA125 (Hz)	66.9 ± 9.9	74.1 ± 14.5	0.247	0.025	−0.556–0.633
MMG AMP at JA75 (m∙s^−2^)	0.44 ± 0.20	0.41 ± 0.19	0.550	0.791	0.326–0.949
MMG AMP at JA125 (m∙s^−2^)	0.49 ± 0.16	0.47 ± 0.18	0.843	0.158	−0.641–0.733
MMG MPF at JA75 (Hz)	21.1 ± 4.7	23.3 ± 6.4	0.089	0.774	0.276–0.944
MMG MPF at JA125 (Hz)	27.7 ± 7.8	24.8 ± 7.1	0.277	0.491	−0.148–0.853

P = Alpha from the ANOVA (2,1 model) for systematic error; ICC = intraclass correlation coefficient; ICC95% = ICC 95% confidence interval; EMG AMP = electromyographic amplitude; EMG MPF = electromyographic mean power frequency; MMG AMP = mechanomyographic amplitude; MMG MPF = mechanomyographic mean power frequency.

## Data Availability

The data sets generated during and/or analyzed during the present study are available from the corresponding author upon reasonable request.
